# Challenges of Applying the RANO-BM Criteria for Characterization of Brain Metastases Treatment Response

**DOI:** 10.3390/curroncol33020077

**Published:** 2026-01-28

**Authors:** Tatiana Kashtanova, Naren Ramakrishna

**Affiliations:** 1 Department of Computer Science, Johns Hopkins University, Baltimore, MD 21218, USA; 2 Department of Radiation Oncology, Orlando Health Cancer Institute, Orlando, FL 32806, USA; 3 College of Medicine, University of Central Florida, Orlando, FL 32827, USA

**Keywords:** brain metastases, response assessment, RANO-BM

## Abstract

The goal of brain metastasis therapy is to reduce the risk of intracranial disease progression and to minimize treatment-related adverse effects and loss of neurologic function without compromising extracranial disease control. A response assessment system plays a critical role in the comparative evaluation of therapeutic strategies in clinical trials and in routine patient care. Since 2015, the RANO-BM criteria have become a standard schema for evaluating brain metastases treatment response, providing uniform definitions and methodology particularly practical in prospective clinical trials of systemic therapy. There have been a variety of modifications and additions to the original guidelines proposed to improve their utility for brain metastases response assessment, including lowering the measurable disease size threshold, optimizing disease progression metrics, and employing tumor volumetric analysis using automated measurement tools. However, despite these enhancements, the criteria display limitations in selected clinical scenarios. This article provides a detailed overview of these limitations and their corresponding clinical contexts and concludes with a discussion of approaches which may aid in the development of a more comprehensive brain metastases response assessment system.

## Simple Summary

Brain metastases treatment response assessment is an essential component of clinical trials and standard patient care. Therefore, its scope, sensitivity, and adaptability to a wide range of clinical scenarios are of great importance. This work provides a comprehensive review of the current standard brain metastases response assessment methodology proposed by the RANO-BM (Response Assessment in Neuro-Oncology working group for Brain Metastases) and its limitations when applied to specific clinical situations. The review is complemented by a discussion of possible modifications to the RANO-BM guidelines which may contribute to the development and refinement of future brain metastases response assessment systems.

## 1. Introduction

A major cause of morbidity and mortality for cancer patients is the development of brain metastatic disease [1]. Brain metastases are the most common intracranial malignancy and may ultimately develop in up to 40% of patients with solid tumors [2,3,4,5]. The treatment paradigms for brain metastases treatment have grown increasingly individualized and complex and often include surgery, whole-brain radiation therapy (WBRT), stereotactic radiosurgery (SRS), fractionated stereotactic radiotherapy (FSRT), and systemic agents with central nervous system (CNS) activity [3,4,5,6]. In addition, the utility of new modalities such as tumor-treating fields is under evaluation [7,8]. The assessment of brain metastases treatment response is challenging because it requires an analysis of the dynamic time-dependent change in treated tumor size, the appearance of new brain metastatic lesions, and the progression of lesions outside of the treated field—distant brain failure (DBF) events [9]. Furthermore, the assessment may be complicated by the potential development of radiation necrosis, immune-mediated post-treatment inflammation, diffuse or leptomeningeal disease [9,10,11], as well as the interplay between separate radiotherapy treatments and treatments with surgery and systemic therapies which patients may undergo sequentially or concurrently [5,12,13,14]. Brain metastases response assessment criteria are a fundamental component of clinical trials, and for patients who are not enrolled in trials, the criteria play an equally important role in determining optimal treatment and patient prognosis.

A widely used schema for evaluating brain metastases treatment response is the RANO-BM criteria [9] proposed by the Response Assessment in Neuro-Oncology Bain Metastases working group in 2015 to address the need for uniform definitions and standards in brain metastases response assessment. The criteria use unidimensional tumor measurements, monitor the change in size of the ≤5 largest measurable (“target”) lesions, qualitatively analyze the behavior of other (“non-target”) lesions, incorporate patient clinical status based on the Karnofsky performance score (KPS), and account for corticosteroid usage. Depending on these parameters, the patient overall response (Table 1) is categorized as Complete Response (CR), Partial Response (PR), Stable Disease (SD), or Progressive Disease (PD). The criteria define a measurable lesion as a contrast-enhancing lesion with the longest diameter of ≥10 mm and the greatest perpendicular diameter of ≥5 mm. RANO-BM allows for lowering the measurable disease size threshold while emphasizing the importance of accurate and reproducible measurements [9,11]. Considering that MRI slice thickness might affect tumor measurement and lesion detection [15], especially in the case of small brain metastases, the criteria recommend using thin-slice MRI technique and confirming new lesion development on repeated images. In addition, the group provides guidelines on interpreting pseudo-progression related to immunotherapy and radiosurgery as well as volumetric tumor response analysis. The overall assessment methodology was meant to be validated via the analysis of historical data [11].

Several research groups have examined the application and optimization of the RANO-BM criteria to brain metastasis data by exploring the impact of lowering the measurable disease size threshold, changing disease progression metrics, using automated tumor size measurements, and considering tumor volume instead of its longest dimension. Thus, the study by Qian et al. [12] on brain metastases treated in the setting of systemic immunotherapy suggested the use of a 5 mm measurable lesion longest diameter threshold instead of 10 mm to allow for more evaluable patients when using MRI slice thickness ≤ 2 mm. Iorio-Morin et al. [13] utilized a 5 mm measurable lesion threshold when assessing the safety and efficacy of repeated stereotactic radiosurgery. Douri et al. [16] evaluated outcomes following repeated SRS for locally recurrent brain metastases and suggested defining tumor progression as a 2.5 mm absolute diameter increase relative to nadir instead of a 20% relative percentage increase to improve analysis sensitivity. Given that the estimation of tumor size changes is a critical element in response assessment, considerable effort has been devoted to the development of automatic lesion measurement algorithms [17,18,19] and the use of medical image analysis software (e.g., 3D Slicer v. 4.5.0, OncoTREAT v. 1.6, Jazz 2023) [5,20,21,22,23,24] to facilitate tumor response assessment based on conventional unidimensional lesion measurements [19,22,23,24] and volume [5,17,18,19,20,21,23,24]. Over the last two decades, the importance of volumetric tumor evaluation has been challenged in different tumor response assessment criteria, and the opinions have been mixed. While some studies underscored the superiority of tumor volume in minimizing the intra- and interobserver variability of tumor measurements [21] and lesion response characterization [24], other studies [5,25,26,27] did not indicate a definite advantage. As of today, the volumetric assessment is technically challenging to implement for routine clinical use and trials, and its incremental benefit to response assessment is still being determined.

Despite the ongoing validation and optimization of the RANO-BM guidelines, the criteria exhibit inherent limitations when applied to specific real-world clinical scenarios. This article provides a detailed overview of these limitations in the corresponding clinical contexts and concludes with a discussion of possible approaches which may aid in the development of a more comprehensive brain metastases response assessment system.

## 2. Single Modality Response Assessment

**Figure 1:**
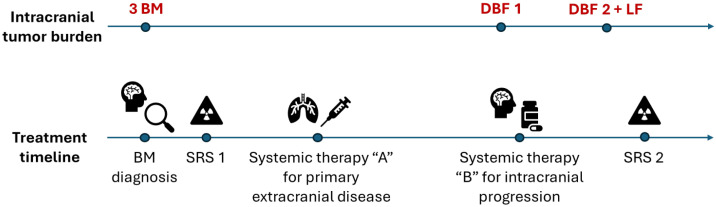
Hypothetical scenario of a breast cancer patient with three brain metastases undergoing multiple treatments: BM—brain metastases, DBF—distant brain failure, LF—local failure, SRS—stereotactic radiosurgery.

The RANO-BM criteria were designed for use in prospective clinical trials and were especially optimized for trials of systemic therapy. The criteria do not address response assessment in the setting of sequential treatments (e.g., repeated SRS) or concurrent mixed modality therapies (e.g., SRS with systemic therapy) [12,13,14] even though these scenarios represent the standard of care of modern brain metastases management and may be used in prospective clinical trials [7]. Figure 1 depicts a common clinical scenario where a patient with newly diagnosed breast cancer develops brain metastases and undergoes multiple treatments.

The symptomatic brain metastases are targeted with SRS1 and the primary disease in the breast is addressed with systemic therapy “A”, which does not have CNS penetration but is the first-line treatment for the extracranial cancer. Later, a new asymptomatic brain metastasis appears—the 1st distant brain failure event (DBF1). The physician changes systemic therapy “A” to systemic therapy “B” with CNS penetration/activity. A few months later, while receiving systemic therapy “B”, the patient presents with another new brain lesion (DBF2) and a progression of previously treated brain metastases. If the local failure (LF) involves one or more metastases previously treated with SRS1, then it reflects the local control ability of SRS1 together with the local control of systemic therapy “B”. However, if the local failure is of the metastasis treated with systemic therapy “B” (DBF1), then it reflects the local control of that treatment only. The physician prescribes SRS2 treatment for the new and progressive metastases and determines potential next systemic therapies based on intracranial and extracranial disease control. In this scenario, the therapies could have been reordered or combined. For example, SRS2 could have been used at DBF1, and systemic therapy “B” used either concurrently or at DBF2 + LF. Comparing different treatment sequences and attributing local and distant control to the appropriate local and/or systemic therapy in a large population requires the development of new assessment methodologies. In the setting of brain metastases prospective clinical trials, a RANO-BM response assessment could have taken place in distinct periods of the overall patient care, e.g., from SRS1 through DBF1, from systemic therapy “B” to DBF2 + LF, or following SRS2, trying to evaluate the success of each treatment modality separately, but it is not designed to assess the composite response of a sequence of therapies accounting for their individual impact and interactions.

## 3. The Sum of Target Lesion Diameters

### 3.1. Individual Lesion Progression

**Figure 2:**
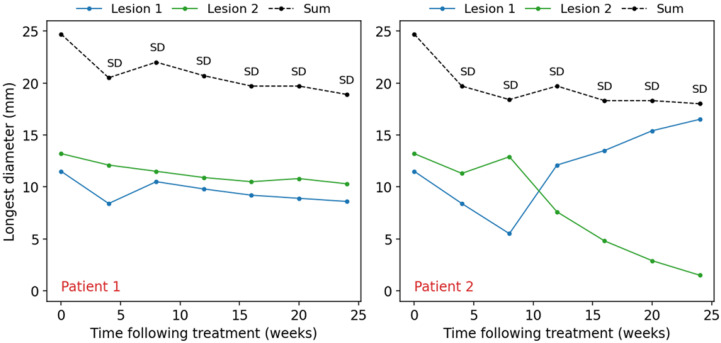
Hypothetical scenario of two patients: blue and green solid lines represent the changes in the longest diameters of two brain metastatic lesions following initial treatment; dashed black lines represent the changes in the sum of the lesion diameters; SD—stable disease response category.

RANO-BM characterizes the response of target lesions based on the sum of their longest diameters to simplify the assessment of multiple lesions. Unless the sum increases by 20% relative to the smallest sum on study, the criteria do not consider the change in size of individual lesions. This is problematic as it might conceal a lesion progression. For example, consider a situation where two patients, each with two brain metastases, receive treatment with either local or systemic therapy (Figure 2). In Patient 1, both lesions get smaller, and their sum decreases by <30% relative to baseline such that the SD response category is fairly assigned to all assessment time points following the treatment. In Patient 2, one lesion shrinks by >80% as compared to its initial size while another lesion grows threefold relative to its smallest dimension. As the sum of the two lesions decreases by <30% relative to baseline and the patient does not exhibit significant neurological symptoms, the overall patient response is characterized as SD. While the response of both patients is SD according to RANO-BM, it is apparent, however, that Patient 2 has had progression in one of the two lesions, which reflects a poorer outcome and might necessitate additional clinical treatment. Thus, while the use of the sum of lesion diameters in RANO-BM simplifies response assessment, it may mask differences in individual outcomes that are clinically significant. In this example, a response assessment tool that is able to detect the discrepancy between per lesion versus combined overall response would alert the clinician to perform a deeper response assessment analysis.

### 3.2. Individual Lesion Control

**Figure 3:**
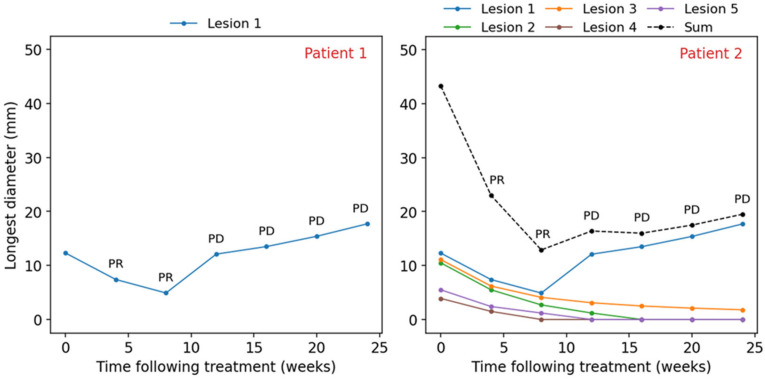
Hypothetical scenario of two patients: solid lines represent the changes in the longest diameters of brain metastatic lesions following treatment; dashed black line represents the changes in the sum of the lesion diameters; PR—partial response category, PD—progressive disease response category.

The sum of target lesion diameters might also obscure the underlying individual lesion control. Consider a situation where one patient is diagnosed with one brain metastasis and another patient is diagnosed with five brain metastases (Figure 3). Both patients receive the same regimen of SRS and/or systemic therapy. Following the treatment, the lesion in Patient 1 shrinks initially and then starts growing by more than threefold relative to its smallest dimension on study, which gets characterized as PD. In Patient 2, one lesion has a similar progressive behavior while the other four lesions exhibit either complete or partial response following the treatment. Since the sum of the target lesions in Patient 2 increases by >20% relative to nadir and one lesion grows by >5 mm, the overall patient response is PD according to RANO-BM. However, the progressive disease category assigned to Patient 2 does not reflect the fact that the treatment was successful for four out of five lesions, which would be of importance to both the physician and the patient. In the RANO-BM assessment, if a patient has only one lesion, the overall response is equal to the individual lesion response; however, if a patient has several lesions, then the overall response is determined by the changes in the sum of the lesion diameters, which might be insensitive to a single lesion progression or conceal its successful management. Thus, in the context of clinical trials, the limitation introduces a bias when comparing treatment responses in patients with one vs. several brain metastases responding to the treatment differently. It would be advantageous for the response assessment system to at minimum distinguish patients in whom PD represents a mixed response and ideally to indicate the fraction of lesions contributing to a particular response category (e.g., 4/5 PR and 1/5 PD) to allow for a more informed clinical analysis.

## 4. PD Category for DBF

### 4.1. DBF Extent

**Figure 4:**
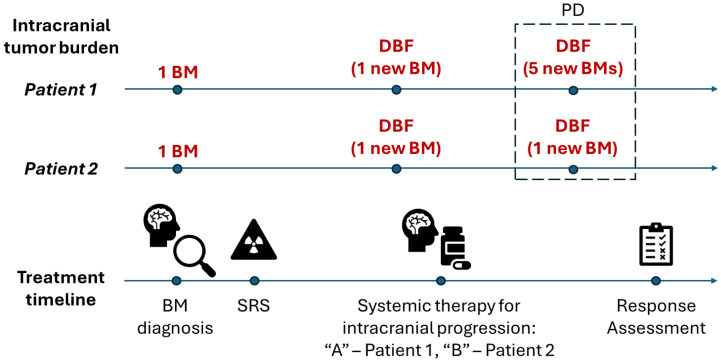
Hypothetical scenario of two patients with brain metastases undergoing different treatments. The patients exhibit the same intracranial tumor burden before systemic therapy: BM—brain metastasis, DBF—distant brain failure, PD—progressive disease response category, SRS—stereotactic radiosurgery.

In the case of systemic therapy trials, the unequivocal appearance of new brain metastases is characterized as disease progression [9]. However, there might be different degrees of distant brain failure which are not reflected in the PD response category but might be of clinical significance. In the hypothetical scenario illustrated in Figure 4, two patients are diagnosed with one brain metastasis and receive SRS. On serial MRI follow-up, Patient 1 develops one new brain metastasis which gets treated with systemic therapy “A”. Later, the patient presents with five new brain metastases confirmed on subsequent scans. Patient 2 also develops one new brain lesion after SRS and receives systemic therapy “B” for intracranial disease control. Later, Patient 2 gets diagnosed with another unequivocal brain lesion. If a RANO-BM response assessment is carried out to compare the efficacy of systemic therapy “A” vs. “B”, the responses of both patients would be labeled as PD with equal time to intracranial progression. However, even though the patients had the same intracranial tumor burden before the systemic therapies, the extent of their subsequent tumor progression was dissimilar (five new lesions vs. one new lesion). Since the number of brain metastases has been shown to be important in predicting patient survival [1,28,29,30,31], quantifying distant brain failure at the time of progression in clinical trials could provide additional insights into the treatment efficacy of different therapies.

**Figure 5:**
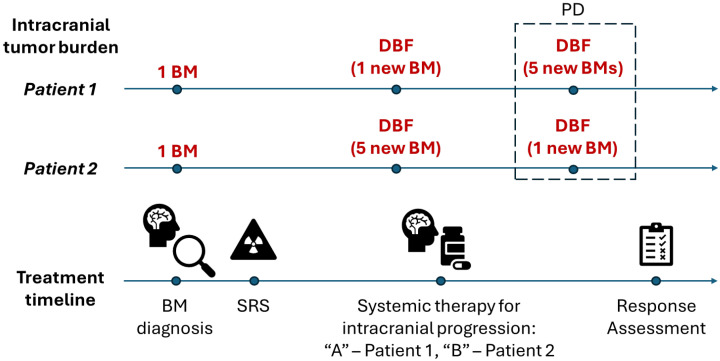
Hypothetical scenario of two patients with brain metastases undergoing different treatments. The patients exhibit dissimilar intracranial tumor burden before systemic therapy: BM—brain metastasis, DBF—distant brain failure, PD—progressive disease response category, SRS—stereotactic radiosurgery.

Now consider the situation where both patients have varied intracranial tumor burdens before the start of systemic therapies “A” and “B” (Figure 5). For example, Patient 1 presents with one new lesion after SRS and Patient 2 presents with five new lesions after SRS. Thus, with respect to systemic therapy “A”, Patient 1 starts with one lesion and develops five new lesions. With respect to systemic therapy “B”, Patient 2 starts with five lesions and develops one new lesion. It is apparent that the initial and subsequent intracranial tumor burden for systemic therapy response assessment are different. However, this is not accounted for in the RANO-BM assessment, and the response of both patients is reported as PD, once again potentially masking inherent differences in the treatment response.

The volume of brain metastases comprising DBF events among different patients/treatment arms is also not directly considered in RANO-BM even though clinical data suggest that DBF volume may be associated with local control and patient prognosis [28,30,31,32]. For example, in the latter scenario, if the five new brain metastases in Patient 1 are small, they may be easily addressed with SRS, whereas the single new metastasis in Patient 2, if large, may be much more symptomatic and require surgical resection. This underscores the limitation of using overall intracranial progression as a binary endpoint as it is currently defined while not including the number and/or volume of DBF events characterizing the progression extent.

### 4.2. DBF Rate

**Figure 6:**
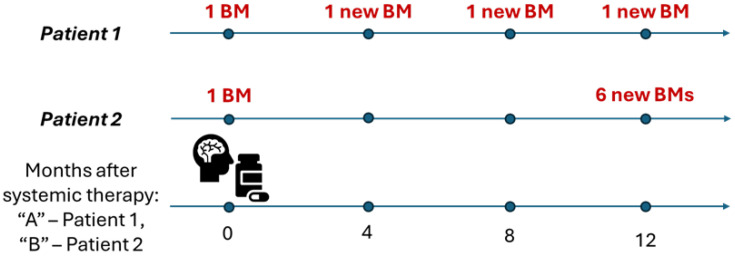
Hypothetical scenario of two patients with a history of new brain metastases following the start of systemic therapy: BM—brain metastasis.

The lack of DBF quantification in the RANO-BM assessment has further implications when ignoring the rate of DBF events. Consider a situation (Figure 6) where two patients, each presenting with a single asymptomatic brain metastasis, are started on systemic therapies “A” and “B”. Patient 1 presents with a new brain metastasis every 4 months over a 12-month follow-up period, resulting in an overall numerical degree of failure of three new brain metastases with a DBF latency of 4 months. Patient 2 presents with six new brain metastases at 12 months, displaying an overall numerical degree of failure of six new brain metastases with a DBF latency of 12 months. According to RANO-BM, the response of Patient 1 would get marked as DBF three times, while the response of Patient 2 would get assigned to the DBF category once, with no consideration for the time elapsed between the events and the extent of the failures. The clinical significance of new intracranial disease depends on the number, volume, and rate of appearance of new lesions. Previous studies have shown that the Brain Metastasis Velocity (BMV) as the numerical rate of new brain metastases development [33] and the volumetric Brain Metastasis Velocity (vBMV) accounting for new lesion volume [34] were strong predictors of patient survival. Therefore, supplementing the RANO-BM DBF (or PD) response category with these parameters would be informative for physicians in evaluating treatment response.

## 5. Patient Clinical Status

The use of KPS as the sole patient clinical status metric in RANO-BM is problematic for brain metastases response assessment where adverse events due to treatment side-effects or failures would likely be better reflected in patient neurological status rather than in their overall performance status [11,14]. While the RANO-BM criteria state that a decrease of KPS from 90–100 points to 70 points or less, a decrease from 80 points or less by at least 20 points, or a decrease from any starting score to 50 points or less would indicate patient clinical deterioration, unless the decrease is associated with comorbid conditions, treatment-induced toxicity, or corticosteroid dose changes [9], the indirect metric is difficult to use retrospectively and it does not provide information specific to patient neurological symptoms, which are of interest to clinicians treating brain metastatic disease. It would be more meaningful to define patient neurologic clinical status based on their brain-specific symptoms (BSS), which, in the case of many retrospective clinical trials, could be determined via a review of patient medical records created at the time of treatments and follow-up visits. The BSS score could be based on the severity of general neurologic dysfunction according to Common Terminology Criteria for Adverse Events (CTCAE) [35], as presented in Table 2.

**Figure 7:**
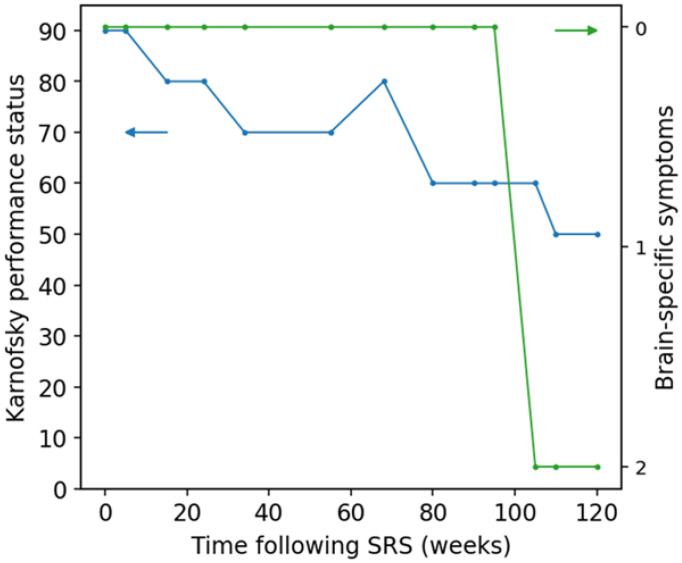
Hypothetical scenario of a patient with primary lung and metastatic brain cancer. The plot indicates the fluctuations of patient Karnofsky performance score (blue) and brain-specific symptoms (green) following stereotactic radiosurgery (SRS) for brain metastases. The blue and green arrows indicate the axes corresponding to the respective lines.

As an example of the potential disadvantage of relying on the KPS score exclusively, consider a hypothetical situation (Figure 7) where a patient with primary lung cancer develops brain metastatic disease and receives SRS. Following the treatment, the patient KPS score fluctuates considerably due to shortness of breath, back pain, and limited mobility secondary to pathologic hip fracture. At the same time, the patient BSS score remains stable for over 100 weeks post-SRS. When analyzing retrospective/historical data, the interpretation of KPS score variations across sequential assessments is challenging and sometimes impossible due to a lack of information and thus might result in incorrect treatment response categorization (e.g., false PD not reflecting treatment failure), which could be avoided when using the BSS score. Moreover, the BSS metric represents the true patient neurological symptom and functional status, which cannot be inferred reliably from the overall functional status given by KPS.

Recently, the Neurological Assessment of Neuro Oncology (NANO) criteria [36,37] were proposed to overcome the limitation of KPS in quantifying the clinical condition of patients with brain tumors. The criteria are based on a complex patient neurological examination that must be completed at the time of patient assessment, which is suitable for prospective clinical trials but impossible to apply in retrospective studies. The BSS metric, on the other hand, requires less information than NANO, allowing for the retrospective evaluation of post-treatment patient neurological symptoms. In the setting of prospective clinical trials, the simple BSS metric may supplement the extensive NANO criteria.

## 6. Target/Non-Target Lesion Designation

### 6.1. Initial Lesion Designation

In the RANO-BM criteria, target/non-target lesion designation is based on the measurable disease size threshold (≥10 mm in the longest dimension) and the limit on the number of target lesions (5 lesions maximum) [9]. Considering that brain metastases of <5 mm in the longest dimension are often treated with SRS [20,38] and/or systemic therapy, the RANO-BM measurable disease size threshold makes many treated brain lesions non-target and reduces the number of patients eligible for response assessment due to a lack of measurable disease. As was mentioned previously, this concern was raised and some groups [12,13] recommended the 5 mm measurable disease size threshold as the criteria limitation countermeasure while still maintaining the possibility of accurate and reproducible measurements when using MRI scans with ≤2 mm slice thickness. However, the RANO-BM constraint on the number of target lesions might also result in many treated measurable and non-measurable lesions becoming non-target because it is a common clinical scenario nowadays to treat 20 or more brain metastases in a single radiosurgery session [39], or with WBRT, and/or systemic therapy. While the rationale for five target lesions was based on the historical evolution from RECIST and the consideration of simplifying clinical trial analysis, the discordance between the treatment “targets” and the response assessment “targets” in the context of brain metastases seems arbitrary and does not promote response assessment accuracy and consistency within study populations and between clinical trials because all non-target lesions are subjected to a qualitative evaluation in RANO-BM where unequivocal progression is up to individual physician judgement.

### 6.2. Sequential Lesion Designation

According to RANO-BM, the unequivocal appearance of new lesions is associated with disease progression (Table 1). However, there is no guidance on how to evaluate the new lesions at subsequent follow-up assessments after declaring DBF/PD at their initial appearance in the setting of focal radiation therapy or sequential systemic therapies, considering that the newly appeared lesions may later be treated successfully. Thus, when patients receive therapy at the time of initial presentation with brain metastases and are followed after progression due to new lesions that were also subsequently treated, should those new lesions always be considered as non-target or would clinical response assessment be better served if they were evaluated as target? Similarly, should brain metastases on initial MRI be designated to target/non-target categories per RANO-BM or would it be better to have the assessment “targets” be the same as the treatment “targets”, as was discussed in the previous section?

**Figure 8:**
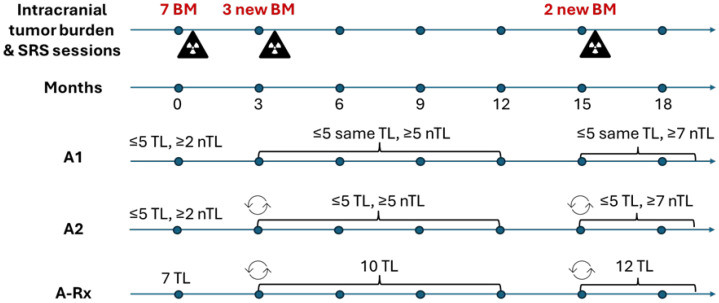
Hypothetical scenario of a patient with brain metastases undergoing sequential radiotherapy sessions: BM—brain metastases, SRS—stereotactic radiosurgery, TL—target lesions, nTL—non-target lesions. The cycle sign indicates the reset of the minimum sum and the baseline sum of the longest diameters of target lesions. The radiation sign indicates stereotactic radiosurgery (SRS) sessions. A1, A2, and A-Rx are alternative approaches to target/non-target brain metastases designation.

The top of Figure 8 illustrates the clinical course of a hypothetical patient who presents with seven brain metastases and undergoes SRS for all of them while receiving systemic therapy. Serial imaging at 3-month-intervals is performed to assess treatment response. At the first follow-up assessment, three new brain metastases are noticed and scheduled for SRS. The response of all lesions is monitored, and at the 15th month after initial diagnosis, two other new brain metastases are reported. The patient receives additional SRS treatment and the response assessment, now involving 12 lesions, continues.

Consider three alternative approaches to designating brain metastases in this response assessment while reporting a DBF/PD event at the initial appearance of new lesions. In the first approach (A1), ≤5 target lesions are selected on the patient’s initial MRI, as recommended by RANO-BM, and other lesions, including those appearing subsequently, are evaluated as non-target. In the second approach (A2), ≤5 target lesions are initially chosen in accordance with RANO-BM. At the time of a DBF event, if the size of a new lesion meets the measurable disease size threshold and the patient has <5 target lesions at the current assessment, the new lesion is added to the target lesions. If the new lesion is measurable and the patient already has five target lesions, then the new lesion replaces a target lesion if its longest diameter is greater than the longest diameter of the target lesion at the current assessment. If several new lesions appear simultaneously, then the replacement procedure is repeated for each new measurable lesion starting from the largest one. The response of the target lesions that have been replaced by new lesions is analyzed qualitatively at subsequent assessments. If the new lesion is non-measurable or if it is smaller than the target lesions, it is evaluated as non-target. Lastly, in the treatment-based approach (A-Rx), there is no limit on the number of target lesions and there is no measurable disease size threshold: all treated brain metastases are considered as target lesions starting from the date when their pre-treatment MRI is obtained. Until then, the lesions are evaluated as non-target. In A2 and A-Rx methods, the minimum sum and the baseline sum of the longest diameters of target lesions are reset whenever the composition of the target lesions changes (Figure 8).

Considering that non-target lesions are assessed qualitatively, the sum of measured lesion diameters might be insensitive to a single lesion progression (Section 2), and since the target lesions composition can change in A2 and A-Rx methods, the analysis might result in different response assessment categories, thus underscoring the need for establishing a uniform approach to brain metastases designation, especially involving new treated lesions.

The A1 approach is the simplest and requires the consideration of fewer lesion measurements, but it is problematic in the setting of sequential SRS/FSRT sessions and/or systemic therapies as the metastases selected for treatment are evaluated as non-target by this method. The A2 approach involves complex operations of measurement substitution and/or addition, which can be unwieldy to apply in large brain metastases datasets. The A-Rx approach requires measurements of all lesions, but it may be more clinically intuitive because it regards every treated lesion as a target lesion from the time of treatment. Furthermore, it provides a uniform assessment framework for both the small and large brain metastases that undergo treatment.

## 7. Discussion

The goal of brain metastasis therapy is to reduce the risk of intracranial disease progression and to minimize treatment-related adverse effects and the loss of neurologic function without compromising extracranial disease control. A response assessment system is critical for the comparative evaluation of therapeutic strategies in prospective clinical trials, retrospective analysis, and routine practice.

The RANO-BM criteria have been proven to be a robust and flexible framework for evaluating brain metastasis response, particularly in the setting of prospective clinical trials of systemic therapy. Previous studies have proposed a variety of modifications and additions to the original criteria to improve their utility, including lowering the measurable disease size threshold, optimizing disease progression metrics, and employing tumor volumetric analysis using automated measurement tools. However, despite these enhancements, the criteria display certain limitations in different clinical scenarios, which may be encountered during the response monitoring of brain metastasis patients. Table 3 summarizes these limitations, their general clinical impact, and possible future directions to address them.

The majority of brain metastasis patients are managed with a combination of systemic and local therapies administrated sequentially and/or concurrently. While local therapies such as SRS/FSRT are expected to predominantly affect local control, systemic therapies may impact both local control and DBF. In patients undergoing mixed-modality treatments, attributing local and distant brain control among therapies might facilitate the identification of favorable treatment sequences and combinations among large populations (Section 2). While RANO-BM likely did not intend to distinguish between the effects of different modalities, there is a need for such a methodology considering the current practice of brain metastases management, which typically involves more than one therapy.

The use of the sum of lesion diameters in RANO-BM to determine the intracranial control was adopted to simplify response assessment for multiple lesions. However, under select circumstances, this approach may mask individual lesion progression or conceal its successful management (Section 3), thereby potentially compromising the validity of the endpoint. The sum also introduces a bias when comparing treatment responses in patients with one vs. several brain metastases displaying variable treatment responses. For an accurate characterization of the heterogeneous effects of treatment on individual lesions, it may be helpful to evaluate each lesion response independently and report the fraction or percentage of lesions contributing to a particular response category when comparing outcomes between patients or populations, respectively.

After the use of upfront local therapy such as SRS/FSRT, the predominant benefit of subsequent therapies may be a reduction in distant brain failure. The RANO-BM criteria for intracranial progression do not quantify DBF at individual assessment time points (Section 4.1) and the overall follow-up period (Section 4.2), even though the number, volume, and rate of new metastases have been associated with overall patient survival and are likely to represent an important indicator of treatment efficacy. It is also useful to separately characterize the local and distant components contributing to intracranial progression, even in the context of systemic therapy. Future methodologies should incorporate these considerations into the response assessment scheme.

Given that brain metastasis progression usually results in neurologic effects, the use of KPS as the sole patient clinical status metric in RANO-BM is problematic (Section 5) as it does not directly reflect patient neurological status and might contribute to false PD categories from non-CNS disease progression in retrospective studies. The proposed BSS metric might be more suitable for post-treatment patient neurological symptoms evaluation, and it could be used in both prospective and retrospective research. Since the metric is novel, validation studies are required to assess its reproducibility and reliability in relation to established tools such as NANO which also quantify brain tumor patient neurological function.

Finally, the RANO-BM criteria call for the selection of up to five target lesions, with the remainder to be considered non-target. This is difficult to rationalize when an increasing proportion of patients are being treated for large numbers (≥20) of brain metastases with local (e.g., SRS/FSRT) and systemic therapies. The target/non-target dichotomy becomes awkward to implement in modern clinical trials and results in the discordance between the treatment “targets” and the response assessment “targets” (Section 6.1), which does not promote response assessment accuracy and consistency within study populations and between clinical trials because all non-target lesions are subjected to a less precise qualitative evaluation based on individual physician judgement. This is further exacerbated by a lack of guidance on characterizing new lesions at subsequent assessments when patients are followed despite DBF events and the new lesions get subsequently treated (Section 6.2). The proposed A-Rx approach for target/non-target lesion designation might be more clinically intuitive and could provide a uniform assessment framework for all treated brain metastases, including newly developed ones. However, considering that the selection of brain lesions for treatment may vary in clinical practice, outcomes assessed using the A-Rx approach might differ if the assignment of lesions into target and non-target groups diverges. Nevertheless, the problem might be considerably less prominent compared to the RANO-BM lesion designation methodology where at most five lesions may be target and all other lesions must be assessed qualitatively. Since the A-Rx is a novel metric, further studies are needed to validate its reliability.

## Figures and Tables

**Table 1:** Summary of the RANO-BM patient overall response assessment * [9].

	CR	PR	SD	PD
Target lesions	None	≥30% ↓ in SLD relative to baseline	Not PR, not PD	≥20% ↑ in SLD and ≥5 mm ↑ in ≥1 LD relative to nadir
Non-target lesions	None	Stable or improved	Stable or improved	Unequivocal progression
New lesions	None	None	None	Present
Corticosteroids	None	Stable or decreased	Stable or decreased	Not applicable
Clinical status	Stable or improved	Stable or improved	Stable or improved	Worse
Requirement	All	All	All	Any

* CR—complete response, PR—partial response, SD—stable disease, PD—progressive disease, SLD—sum of longest diameters of target lesions, LD—longest diameter of a target lesion, ↓—decrease, ↑—increase.

**Table 2:** Brain-specific symptoms (BSS) metric for patient clinical status quantification.

BSS Score	Neurologic Symptoms Severity
0	None
1	Mild symptoms not affecting activities of daily living and with no intervention required (e.g., mild headache, paresthesia, weakness)
2	Moderate symptoms affecting instrumental activities of daily living with non-invasive intervention required (e.g., steroids, pain management)
3	Severe symptoms, not immediately life-threatening but limiting self-care activities of daily living and potentially requiring invasive intervention
4	Life-threatening (e.g., complete disability, drastic cognitive decline)
5	Death due to neurologic decline

**Table 3:** Summary of the RANO-BM limitations and potential future directions *.

RANO-BM Limitations	Clinical Impact	Potential Future Directions
Single modality response assessment	Not designed to assess outcomes for mixed modality local/systemic treatments	Distinguish modality-specific effects on local and distant brain control
Sum of target lesion diameters	Might mischaracterize individual lesion control and introduce bias when assessing responses in patients with one vs. multiple brain metastases	Compare per lesion versus combined overall response Report fraction (percentage) of lesions contributing to a particular response category among patients (populations)
PD category for DBF	Does not consider DBF extent and rate in treatment efficacy assessment	Quantify extent of DBF (total number of new lesions and their volume) at the time of progression Supplement DBF (PD) with BMV and/or vBMV parameters
KPS for patient clinical status	Does not directly represent patient neurological status and might lead to false PD responses	Consider the BSS score as an alternative to KPS in prospective and retrospective trials
Target/non-target lesion designation	Might compromise response assessment accuracy and consistency	Consider the A-Rx approach to lesion designation as a uniform assessment framework for all treated brain lesions including new metastases

* DBF—distant brain failure, PD—progressive disease, BMV—brain metastasis velocity, vBMV—volumetric brain metastasis velocity, KPS—Karnofsky performance status, BSS—brain-specific symptoms, A-Rx—an alternative, treatment-based, approach to target/non-target brain metastases designation.

## Data Availability

No new data were created or analyzed in this study.
